# The Oxidative Stress Response Highly Depends on Glucose and Iron Availability in *Aspergillus fumigatus*

**DOI:** 10.3390/jof10030221

**Published:** 2024-03-18

**Authors:** Tamás Emri, Károly Antal, Kinga Varga, Barnabás Csaba Gila, István Pócsi

**Affiliations:** 1 Department of Molecular Biotechnology and Microbiology, Institute of Biotechnology, Faculty of Science and Technology, University of Debrecen, H-4032 Debrecen, Hungarygilabarnabas@gmail.com (B.C.G.); pocsi.istvan@science.unideb.hu (I.P.); 2 HUN-REN–UD Fungal Stress Biology Research Group, H-4032 Debrecen, Hungary; 3 Department of Zoology, Eszterházy Károly Catholic University, H-3300 Eger, Hungary; antalk2@gmail.com; 4 Doctoral School of Nutrition and Food Sciences, University of Debrecen, H-4032 Debrecen, Hungary

**Keywords:** *Aspergillus fumigatus*, carbon limitation, deferiprone, iron homeostasis, oxidative stress, RNA sequencing

## Abstract

Pathogens have to cope with oxidative, iron- and carbon(glucose)-limitation stresses in the human body. To understand how combined iron–carbon limitation alters oxidative stress responses, *Aspergillus fumigatus* was cultured in glucose–peptone or peptone containing media supplemented or not with deferiprone as an iron chelator. Changes in the transcriptome in these cultures were recorded after H_2_O_2_ treatment. Responses to oxidative stress were highly dependent on the availability of glucose and iron. Out of the 16 stress responsive antioxidative enzyme genes, only the *cat2* catalase–peroxidase gene was upregulated in more than two culturing conditions. The transcriptional responses observed in iron metabolism also varied substantially in these cultures. Only extracellular siderophore production appeared important regardless of culturing conditions in oxidative stress protection, while the enhanced synthesis of Fe-S cluster proteins seemed to be crucial for oxidative stress treated iron-limited and fast growing (glucose rich) cultures. Although pathogens and host cells live together in the same place, their culturing conditions (e.g., iron availability or occurrence of oxidative stress) can be different. Therefore, inhibition of a universally important biochemical process, like Fe-S cluster assembly, may selectively inhibit the pathogen growth in vivo and represent a potential target for antifungal therapy.

## 1. Introduction

*Aspergillus fumigatus* is a well-known opportunistic fungal pathogen responsible for a wide range of diseases from allergic reactions to systemic infections. Invasive aspergillosis, as the most life threatening systemic infection frequently caused by *A. fumigatus*, annually affects more than 250,000 people worldwide [1]. The high mortality rate of systemic aspergillosis (40–90%; [2]) as well as the increasing acquired resistance of *A. fumigatus* to antifungals [3,4] accelerated research to elucidate how *A. fumigatus* is able to survive in the human body even under treatment of the infection.

Microbial pathogens have to adapt to ever-changing and often hostile microenvironments within the host [5]. Oxidative stress induced by macrophages and neutrophils is an important part of the many ways in which the human body controls microbial infections [6,7]. Hence, an efficient oxidative stress response is thought to be an important requirement for virulence of microbes including *A. fumigatus* [8]. The increased susceptibility of patients deficient in reactive oxygen species (ROS) production to fungal infection also underlines this view [9]. However, some of the experimental data do not support the hypothesis that decreased oxidative stress tolerance is accompanied by hypovirulence (Table 1). A deficiency of OxrA (oxidation resistance 1 protein) in *A. fumigatus* resulted in hypovirulence and increased susceptibility to ROS [10]. Inactivation of the *aspf3* gene encoding a peroxiredoxin reduced oxidative stress tolerance of *A. fumigatus* which was accompanied with decreased virulence in a mouse model of pulmonary aspergillosis [11]. Deletion of the *pes1* gene encoding a non-ribosomal peptide synthase decreased oxidative stress tolerance and resulted in hypovirulence [12], while the triple deletion of *ppoA*, *ppoB*, and *ppoC* fatty acid oxygenase genes increased both H_2_O_2_ tolerance and virulence [13]. In contrast, although deletion of *sod1* (cytoplasmic CuZn-superoxide dismutase (SOD) gene) or *sod2* (mitochondrial MnSOD gene) but not *sod3* (cytosolic MnSOD gene) increased oxidative (menadione) stress sensitivity of *A. fumigatus*, but even the triple mutant (Δ*sod1*Δ*sod2*Δ*sod3*) had no decreased virulence in murine aspergillosis models [14]. Similarly, deletion of *catA* encoding a conidial catalase decreased H_2_O_2_ tolerance but did not influence virulence of *A. fumigatus* [15]. Deletion of *cat1* or *cat2* encoding mycelial catalase and mycelial catalase–peroxidase, respectively, had no effect on H_2_O_2_ susceptibility and virulence, and even the double-gene-deletion mutant had only slightly increased H_2_O_2_ sensitivity and slightly delayed infection in a rat aspergillosis model [15,16].

The Sho1 sensor and PkcA and MpkA protein kinases as well as Skn7 and Yap1 transcription factors were found to be important in the regulation of the oxidative stress response [17,18,19,20,21]. The absence/reduced activity of these proteins did not alter the virulence of *A. fumigatus*, however increased oxidative stress sensitivity. Moreover, oxidative stress tolerance of *A. fumigatus* is not unique compared to other *Aspergillus* strains with limited medical importance [22,23]. The lack of a link between oxidative stress tolerance and virulence is generally explained by the rather redundant nature of the oxidative stress defense system in *A. fumigatus* [5]. Most in vivo aspergillosis models are based on infection of immunosuppressed rodents. The leukopenic state of the animals may reduce the need for strong oxidative stress tolerance in the fungus, which may also explain why some oxidative stress-sensitive mutants did not show reduced virulence [24].

**Table 1:** In vivo virulence of *A. fumigatus* mutants with altered oxidative stress tolerance.

Genetic Manipulation	Function of the Gene(s)	Phenotype of the Mutant		Reference
Δ*oxrA*	oxidation resistance protein	decreased H_2_O_2_ tolerance	hypovirulence (mice)	[10]
Δ*aspf3*	peroxiredoxin	decreased H_2_O_2_ and O_2_^−^ tolerance	hypovirulence (mice)	[11]
Δ*pes1*	nonribosomal peptide synthetase	decreased H_2_O_2_ and HOCl tolerance	hypovirulence (*Galleria mellonella*)	[12]
Δ*ppoA*, Δ*ppoB*, Δ*ppoC*	fatty acid oxygenases	increased H_2_O_2_ tolerance	hypervirulence (mice)	[13]
Δ*sod1*Δ*sod2*Δ*sod3*	SODs	decreased menadione tolerance	no effect on virulence (mice)	[14]
Δ*catA*	catalase	decreased H_2_O_2_ tolerance	no effect on virulence (rat)	[15]
Δ*cat1,* Δ*cat2*	catalase and catalase-peroxidase	slightly decreased H_2_O_2_ tolerance	slightly delayed infection (rat)	[15]
Δ*sho1*	transmembrane osmosensor	decreased H_2_O_2_ and menadione tolerance	no effect on virulence (mice)	[19]
*pkcA^G579R^*	protein kinase	decreased paraquat and menadione tolerance	no effect on virulence (mice)	[21]
Δ*mpkA*	protein kinase	increased H_2_O_2_ and decreased menadione tolerance	no effect on virulence (mice)	[20]
Δ*skn7*	transcription factor	decreased H_2_O_2_ and *t*-butyl hydroperoxide tolerance	no effect on virulence (mice)	[17]
*DyapA*	transcription factor	decreased H_2_O_2_ and menadione tolerance	no effect on virulence (mice)	[18]

Experimental data demonstrate that a stress response or the susceptibility to a stressor highly depend on culturing conditions like the type and availability of carbon sources [25,26,27], the availability of other nutrients like iron [23,28], or the effect of other stressors acting sequentially or concomitantly [29,30,31]. This condition dependence of stress responses may explain some of the controversial results with the importance of oxidative stress tolerance in virulence, i.e., the in vitro-identified key elements of the oxidative stress response may not be important under conditions occurring in the human body. In line with this, Brantl et al. [32] found that the Aspf3 peroxiredoxin is essential during infection because it can compensate for the loss of iron-dependent antioxidant enzymes under iron-limitation. Here, we investigated how carbon (glucose) and iron limitation typical for microenvironments of the human host [5] influenced the H_2_O_2_-elicited oxidative stress response of *A. fumigatus*. Evaluation of transcriptome data demonstrated that the changes elicited by H_2_O_2_ highly depended on iron and glucose availability, which was particularly true for iron metabolism genes. Properly regulated iron metabolism appears to be important not only for adaptation to iron limitation but also for coping with oxidative stress. Our work supports the view that elements of the iron metabolism (e.g., Fe-S cluster assembly besides siderophore production) can be potential targets of antifungal therapies.

## 2. Materials and Methods

### 2.1. Strain, Culturing Conditions

The *Aspergillus fumigatus* Af293 strain (CBS 101355, received from the CBS-KNAW culture collection; https://wi.knaw.nl/Collection; accessed on 14 March 2024), maintained on Barratt’s minimal agar plates [33], was used for all experiments. Aliquots (100 mL) of Barratt’s minimal broth supplemented with 5 g/L yeast extract were inoculated with 5 × 10^7^ conidia isolated freshly from 6 d agar plate cultures incubated at 37 °C. Submerged cultures were incubated at 37 °C and 220 rpm shaking frequency (approximately 3.7 Hz) for 17 h. Mycelia from 4 × 100 mL of these exponential growth phase cultures were transferred into 12 × 100 mL fresh minimal broth containing 1.52 g/L KH_2_PO_4_, 0.52 g/L MgSO_4_ 4H_2_O and 0.52 g/L KCl (pH 6.5) and 20 g/L glucose, 4 g/L casein peptone (Merck Ltd., Budapest, Hungary) (“glucose + peptone” or “glucose rich” cultures) as the carbon/energy source. These cultures were supplemented with either 0.1 *v*/*v*% Barratt’s trace element solution [33] (“iron supplemented” cultures) or 0.1 *v*/*v*% iron-free Barratt’s trace element solution and 1 mM deferiprone (DFP, Merck Ltd., Budapest, Hungary; “DFP treated” or “iron limited” cultures) as iron chelator [23]. Cultures were incubated for 8 h at 37 °C and 220 rpm, then some of them were treated with 75 mM H_2_O_2_, and samples were taken for RNA isolation 1 h after oxidative stress treatment. The whole experiment was repeated with minimal broth containing only 4 g/L casein peptone (“peptone” or “glucose free” cultures) (instead of 20 g/L glucose and 4 g/L casein peptone).

### 2.2. Measuring H_2_O_2_ Stress Sensitivity of Surface Cultures with Well Diffusion Assay

Minimal agar plates (iron supplemented and iron-limited plates containing glucose and casein peptone or only casein peptone) contained 20 g/L agar and the same components as described with the submerged cultures. To study the oxidative stress sensitivity of the mycelium, conidia were spread out (100 μL suspension containing 2 × 10^7^ conidia/mL) on agar plates and a well (with 6 mm diameter) was made in the center of each plate. After pre-incubation for 24 h at 37 °C, aliquots (50 μL) of 4 M H_2_O_2_ were pipetted into the wells, and cultures were further incubated for 96 h at 37 °C. Stress sensitivity was characterized with the diameter of the inhibition zone.

### 2.3. Detecting Growth, Redox Imbalance and Triacetylfusarinine C Production

The growth of submerged cultures was measured with the increase in the dry cell mass (DCM) as described by Emri et al. [34]. The H_2_O_2_ treatment induced redox imbalance was characterized with the DCF-assay using 2′,7′-dichlorofluorescein staining [35]. Triacetylfusarinine C (TAF-C) production of DFP treated cultures as an indicator of iron limited conditions was examined with thin-layer chromatography (TLC). Aliquots of cell-free fermentation broth were treated with 1 g/L FeCl_3_, and the pH was set to 6.5. Samples (50 μL) were spotted on to TLC Silica gel 60 (Merck Ltd., Budapest, Hungary), and ferri-siderophores were separated with *n*-butanol:acetic acid:water (60:25:15 *v*/*v*%) solvent.

### 2.4. High Throughput RNA Sequencing

Mycelial samples (from three biological replicates of the eight treatments) were lyophilized, and total RNA was isolated using TRI reagent (Merck Ltd., Budapest, Hungary) according to Chomczynski [36]. RNAseq libraries were prepared and sequenced at the Genomic Medicine and Bioinformatic Core Facility, Department of Biochemistry and Molecular Biology, Faculty of Medicine, University of Debrecen, Debrecen, Hungary. For library preparation, a TruSeq RNA Sample preparation kit (Illumina, Praha, Czech Republic) was used according to the manufacturer’s protocol. All library pools were sequenced (single-read 75 bp sequencing) in one lane of a sequencing flow cell on an Illumina HiScan SQ instrument (Illumina, San Diego, CA, USA), and 11–50 million reads/sample were obtained. In the case of each sample, 80–91% of the reads were successfully aligned (hisat2 version 2.1.0; [37]) to the genome of *A. fumigatus* Af293 (Gene Expression Omnibus database; http://www.ncbi.nlm.nih.gov/geo/; accessed on 14 March 2024; accession number: GSE256524). FeatureCounts 2.0.0 [38] was used to generate read counts and RPKM values (reads per kilobase million), while DESeq2 1.24.0 [39] was used to determine differentially expressed genes.

### 2.5. Evaluation of Transcriptome Data

Principal component analysis (PCA) of the transcriptomes based on the rlog values generated by the DESeq2 (version 1.24.0) software was performed using the “prcomp” function of the R project (https://www.r-project.org/; accessed on 14 March 2024). Multidimensional scaling (MDS) of the transcriptional data of selected gene groups based on Euclidian distances calculated with the rlog values was performed with the “dist” (for creating distance matrix) and “cmdscale” R functions (https://www.r-project.org/; accessed on 14 March 2024).

Upregulated and downregulated genes (treatment responsive genes) were defined as differentially expressed genes (adjusted *p*-value < 0.05; Deseq2), where |log_2_FC| > 1. FC (fold change) stands for the number calculated by the DESeq2 (version 1.24.0) when “A” and “B” transcriptomes (“A” vs. “B”) were compared, and “B” was used as the reference transcriptome.

Gene set enrichment analyses (ShiniGo platform; http://bioinformatics.sdstate.edu/go/; accessed on 14 March 2024) were used to characterize the upregulated and downregulated gene sets. Default settings were applied, and hits with a corrected *p*-value < 0.05 were regarded as significantly enriched.

The enrichment of the following gene groups in the upregulated and downregulated gene sets was tested with Fisher’s exact test (*p* < 0.05; “fisher.test” function of R project; https://www.r-project.org/; accessed on 14 March 2024): “Antioxidative enzyme”, “Autophagy related”, “Chitinase”, “Chitine utilization”, “Fe-S cluster assembly”, “Fe-S cluster protein”, “Glucanase”, “Glutathione degradation and synthesis”, “Heme binding protein”, “Heme biosynthesis”, “Iron acquisition” and “Secondary metabolite cluster” genes [26], as well as “Respiration” genes (KEGG pathway database; https://www.genome.jp/pathway/afm00190; accessed on 14 March 2024).

## 3. Results

In order to study the effect of combined iron–carbon limitation on the oxidative stress response of *A. fumigatus* Af293, mycelia from exponentially growing cultures were transferred into glucose–peptone (glucose-rich cultures) or peptone (glucose-free cultures) containing media either supplemented (iron-limited cultures) or not (iron-rich cultures) with 1 mM DFP. After adaptation to culturing conditions, cultures were treated with 75 mM H_2_O_2_.

**Figure 1:**
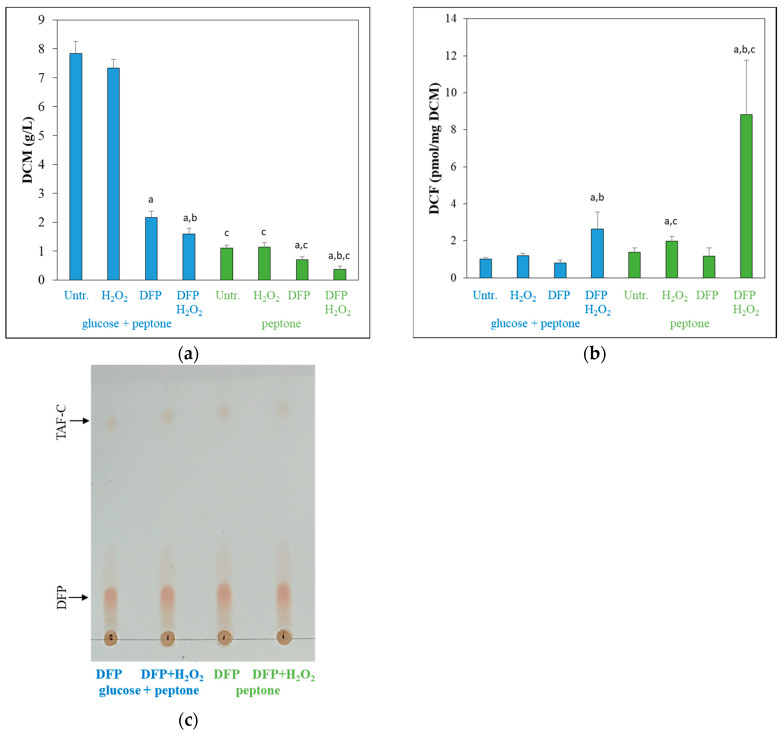
Growth (**a**), redox imbalance detected with the DCF test (**b**), and TAF-C production (**c**) of *A. fumigatus* cultures growing in the presence (blue) or absence (green) of glucose. Mean ± SD calculated from three biological replicates are presented. ^a^ Significant difference (Student’s *t*-test; *p* < 0.05) between the treated (H_2_O_2_, DFP, or DFP + H_2_O_2_) and the appropriate untreated cultures in the presence or absence of glucose. ^b^ Significant difference (Student’s *t*-test; *p* < 0.05) between the DFP + H_2_O_2_ treated and the appropriate DFP treated cultures in the presence or absence of glucose. ^c^ Significant difference (Student’s *t*-test; *p* < 0.05) between the cultures growing in the absence of glucose and the appropriate culture growing in the presence of glucose.

Both glucose withdrawal and DFP treatment reduced the growth of the fungus, but H_2_O_2_ treatment had a growth reduction effect only in DFP-pretreated (iron-limited) cultures (Figure 1a).

In contrast to the observation for peptone, H_2_O_2_ treatment slightly reduced the growth on glucose, but this reduction was not significant (*p* = 0.19, Student’s *t*-test). H_2_O_2_ treatment caused a strong redox imbalance in iron-limited cultures and a less strong imbalance in iron-supplemented cultures in the absence of glucose (Figure 1b). Glucose withdrawal (alone) and DFP treatment had no significant effect on redox homeostasis (Figure 1b). Secretion of TAF-C, an indicator of iron-limitation stress [40], was detected in each DFP-treated culture (Figure 1c).

**Figure 2:**
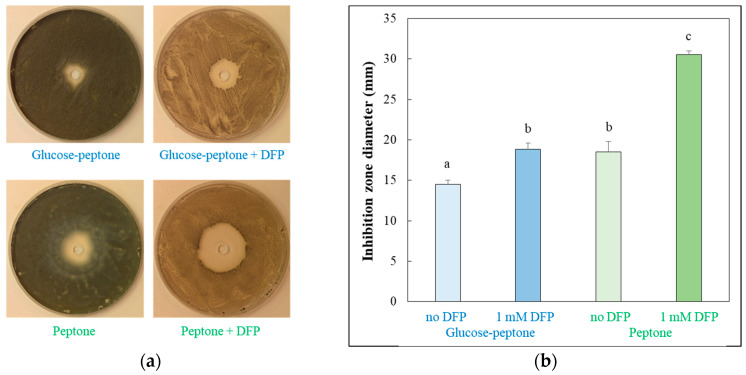
Inhibitory effect of H_2_O_2_ on the growth of *A. fumigatus* in different surface cultures. *A. fumigatus* Af293 was grown at 37 °C for 5 d, on glucose–peptone or peptone agar plates either supplemented or not with 1 mM DFP. H_2_O_2_ (50 μL; 4 mol/L) was added at 1 d. (**a**) Representative photos of the 5 d old cultures. The diameter of the Petri-dish was 85 mm. (**b**) Diameter of the inhibition zone. Means ± SD (*n* = 3) are presented. Different letters indicate a significant difference between the means (one-way ANOVA followed by Tukey post hoc test; *p* < 0.05).

The oxidative stress sensitivity of mycelia was also tested in surface cultures using a well diffusion assay (Figure 2a). Concurring with the results of the DFC test, DFP treatment increased H_2_O_2_ susceptibility of the cultures both in the presence and absence of glucose, and glucose withdrawal also enhanced oxidative stress sensitivity in iron-supplemented cultures (Figure 2).

**Figure 3:**
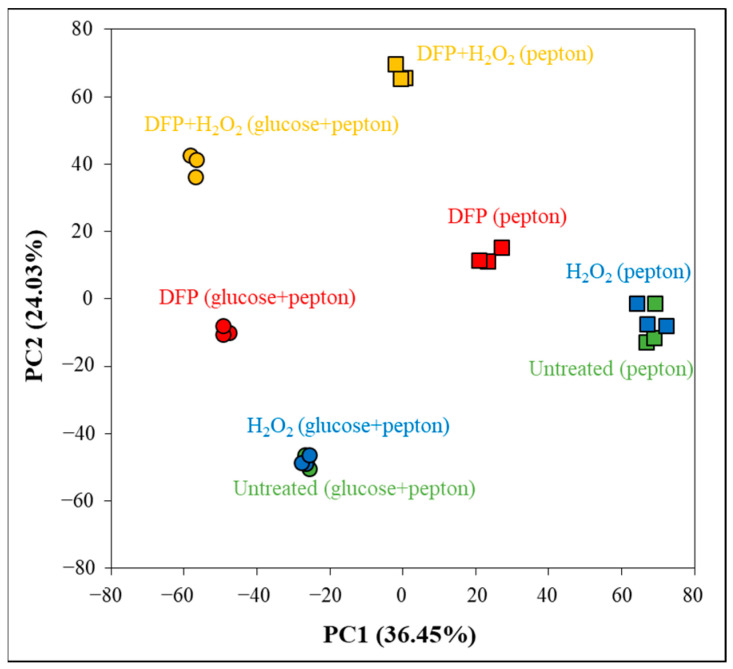
Principal component (PC) analysis of the transcriptomes based on rlog values.

Transcriptomes of all types of cultures were determined using RNA sequencing. Principal component analyses demonstrate that the three biological replicates in each treatment had similar transcriptomes (Figure 3). The presence of glucose and DFP substantially affected the transcriptomes, while the presence of H_2_O_2_ had a substantial effect only in DFP pretreated cultures (Figure 3).

In a previous experiment [26] to test the effects of glucose withdrawal, mycelia from exponentially growing *A. fumigatus* Af293 cultures were transferred into media containing glucose, peptone and NaNO_3_, or only peptone and NaNO_3_. These cultures were incubated for 24 h and 4 h, respectively, and their transcriptomes were determined. In another previous study [28], H_2_O_2_ treatment (3 mM) was applied on untreated and iron-starved *A. fumigatus* Af293 cultures using a glucose and NaNO_3_-containing medium. Iron starvation was initiated by growing the fungus without any iron supplements for 50 h after inoculation with conidia. The iron supplemented cultures were incubated for 33 h to achieve the same glucose content and DCM. In these experiments (similarly to the present study), H_2_O_2_ treatment had a small effect on untreated cultures, but it altered the transcriptome substantially in iron-starved cultures [28]. Because of the very different experimental design (e.g., H_2_O_2_ concentration, induction of iron limitation, composition of the medium, age of the cultures), the observed genome-wide transcriptional changes in the previous experiments [26,28] and in this study did not show a strong positive correlation (Table S1). The Pearson’s correlation coefficients varied between 0.246 (H_2_O_2_ treated vs. untreated cultures) and 0.515 (iron starved vs. untreated cultures) (Table S1).

### 3.1. Effects of Glucose Withdrawal

**Figure 4:**
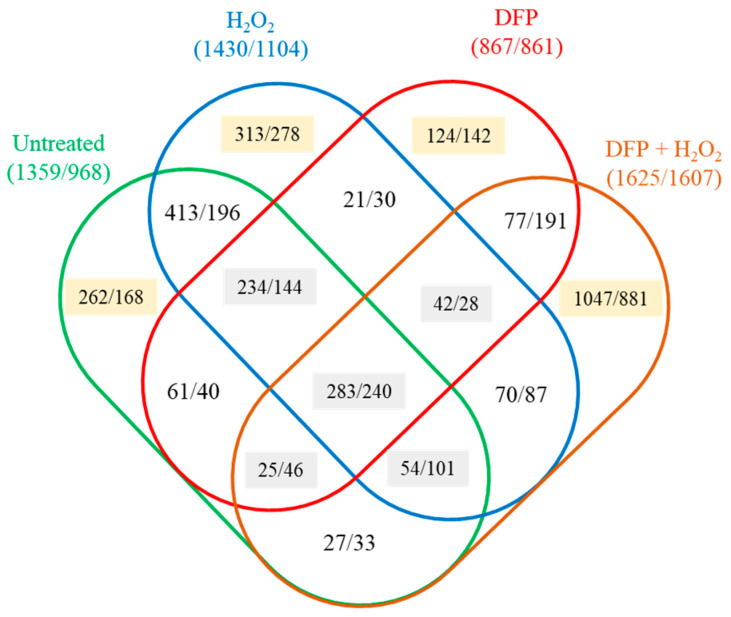
Transcriptional consequences of glucose withdrawal under different culturing conditions. The number of upregulated/downregulated genes are presented. The backgrounds of the numbers of upregulated or downregulated genes encode the number of culturing conditions affected: yellow for one condition, gray for three or more.

Growing on peptone instead of glucose + peptone altered the transcriptomes substantially (Figure 3 and Figure 4). The biggest difference between the transcriptomes was observed under the combined DFP + H_2_O_2_ treatment (Figure 4). Approximately 20% of the treatment (H_2_O_2_, DFP, DFP + H_2_O_2_ treated and untreated) responsive genes showed upregulation (or downregulation) in at least three treatments (Figure 4).

**Figure 5:**
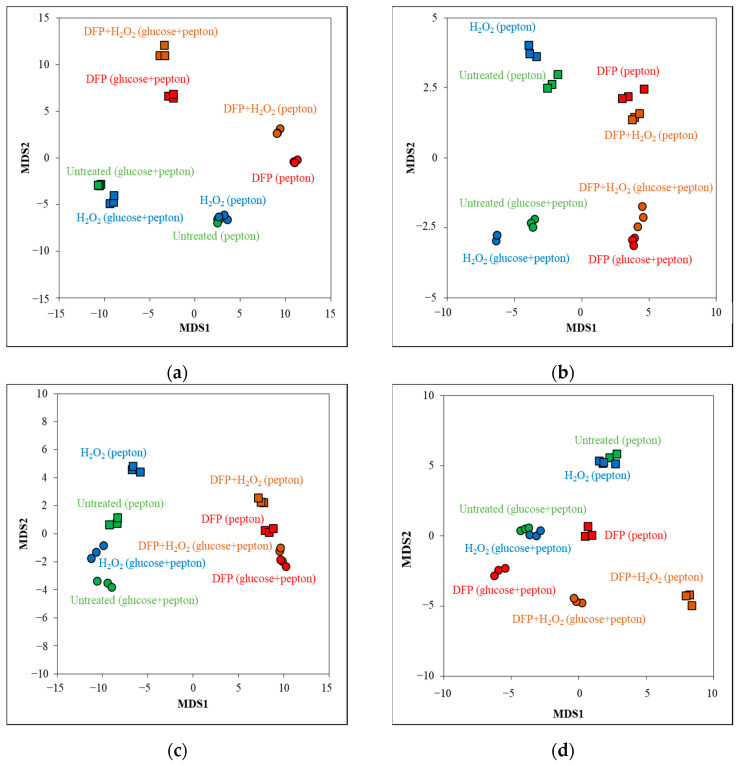
Multidimensional scaling of heme binding protein genes (**a**), Fe-S cluster protein genes (**b**), iron acquisition genes (**c**) and antioxidative enzyme genes (**d**) by their transcriptional activity (rlog values).

In untreated cultures, 1359 genes showed upregulation and 968 genes showed downregulation (Figure 4). The upregulated gene set was enriched in amino acid degradation genes, while the downregulated gene set was enriched in glycolytic genes, indicating that cells replaced glucose with amino acids as a carbon source (Table S2). Cultures showed several properties which were observed earlier in carbon-limited cultures [26]: Fatty acid degradation, plant cell wall polysaccharide degradation and chitin utilization genes as well as gliotoxin and DHN-melanin cluster genes were enriched in the upregulated gene set, while cell wall biogenesis genes were enriched in the downregulated gene set (Table S3). Heme binding protein genes were enriched both in the upregulated and in the downregulated gene sets, demonstrating that glucose-containing and glucose-free cultures use these iron-containing proteins differently (Figure 5a, Table S3).

### 3.2. Effects of DFP Treatment in the Presence and Absence of Glucose

**Figure 6:**
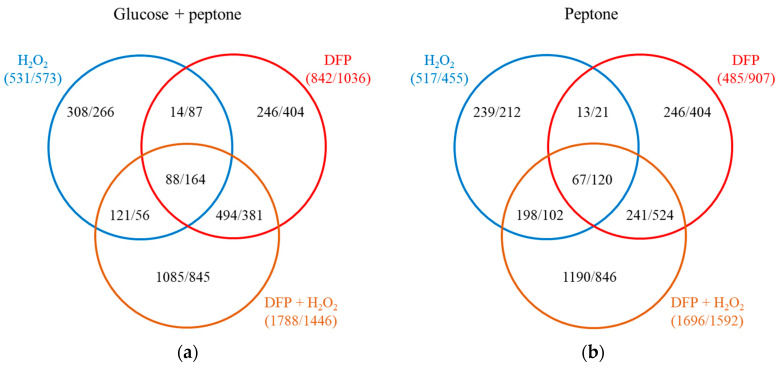
Transcriptional consequences of H_2_O_2_, DFP and DFP + H_2_O_2_ treatments in medium containing glucose + peptone (**a**) or only peptone (**b**) as carbon source. The number of upregulated/downregulated genes are presented.

Adaptation to DFP treatment upregulated 842 genes and downregulated 1036 genes in the presence of glucose, while it upregulated 485 genes and downregulated 907 genes when peptone was the sole carbon source (Figure 6).

**Figure 7:**
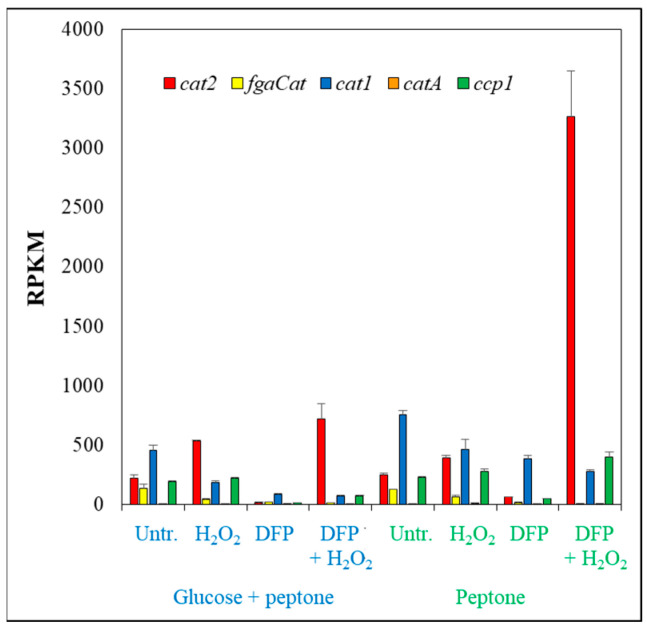
Transcriptional activity of selected catalase and heme peroxidase genes. Means ± SD of three biological replicates are presented. For DEGs, see also Table S3.

The overlaps between the two upregulated or between the two downregulated gene sets were less than 50% (Figure S1), and the Pearson’s correlation coefficient between the two genome-wide transcriptional changes was only 0.433 (Table S1). These data indicate that the two types of cultures responded differently to the same DFP treatment. Nevertheless, there were strong similarities between the two responses when the function of treatment-responsive genes were evaluated. The upregulated gene sets were enriched in iron-acquisition genes including reductive iron-assimilation (RIA; [41]) genes, siderophore biosynthesis and siderophore transport genes [41] as well as *hapX* encoding a positive regulator of iron uptake [41] (while *sreA* encoding the negative regulator of iron uptake [41] was downregulated) in both types of cultures as was expected (Tables S2 and S3). These changes concur with the observed TAF-C production of these cultures (Figure 1c). Upregulation of iron acquisition processes was accompanied with downregulation of several genes encoding iron-containing proteins or processes depending on these proteins (Table S3): TCA cycle, Fe-S cluster protein and heme binding protein genes were enriched in the downregulated gene set irrespectively of the presence of glucose (Table S3). Respiration genes also enriched in the downregulated gene set on glucose + peptone and several respiration genes (all the four succinate dehydrogenase subunit genes, *cycA* cytochrome c gene and two NADH-ubiquinone dehydrogenase subunit genes) showed downregulation on peptone too (Table S3). Regarding heme binding protein genes (Figure 5a), they were enriched not only in the downregulated but also in the upregulated gene set in the absence of glucose (Table S3). The transcriptional activity of these genes was largely dependent on the presence of glucose, in contrast to iron acquisition or Fe-S cluster protein genes, where DFP treatment was the most important factor affecting mRNA abundances (Figure 5b,c). Importantly, downregulated heme binding protein genes included several catalase and heme-peroxidase genes as well (like *cat1*, *cat2*, *catA*, *fgaCat* catalase and *ccp1* cytochrome c peroxidase genes; Figure 7; Ref. [15]), and their downregulation was less prominent on peptone than on glucose + peptone, especially in the case of the *cat1* catalase gene (Figure 7, Table S3). As a consequence, catalase and peroxidase genes were enriched in the upregulated gene set when the transcriptome of the two types of DFP-treated cultures were compared (glucose + peptone, DFP vs. peptone, DFP) (Table S3). Multidimensional scaling of antioxidant enzyme genes also suggested that the transcription of these genes was more dependent on DFP treatment on peptone than on glucose+peptone (Figure 5d). Interestingly, ergosterol biosynthesis genes (although ergosterol biosynthesis also contains iron-dependent steps) were enriched in the upregulated gene sets of both glucose-containing and glucose-free cultures (Table S3).

In DFP-treated glucose + peptone-containing cultures, glucose transport and glycolysis as well as amino acid transport and amino acid catabolism genes were enriched in the downregulated gene set (Tables S2 and S3) in accordance with the hindered growth of the cultures (Figure 1a). In the absence of glucose, the DFP treatment caused a growth reduction (Figure 1a), which was accompanied by the downregulation of chitin utilization genes and “Structural constituent of cell wall” genes as well as the upregulation of “DNA repair complex assembly” genes (Tables S2 and S3). DFP treatment upregulated zinc homeostasis genes (Table S3), and like glucose withdrawal, altered secondary metabolism (Table S3) as well. Upregulation of gliotoxin cluster genes on glucose + peptone and downregulation of DHN-melanin cluster genes both on glucose + peptone and peptone were notable (Table S3).

### 3.3. Oxidative Stress Responses of the Cultures

**Figure 8:**
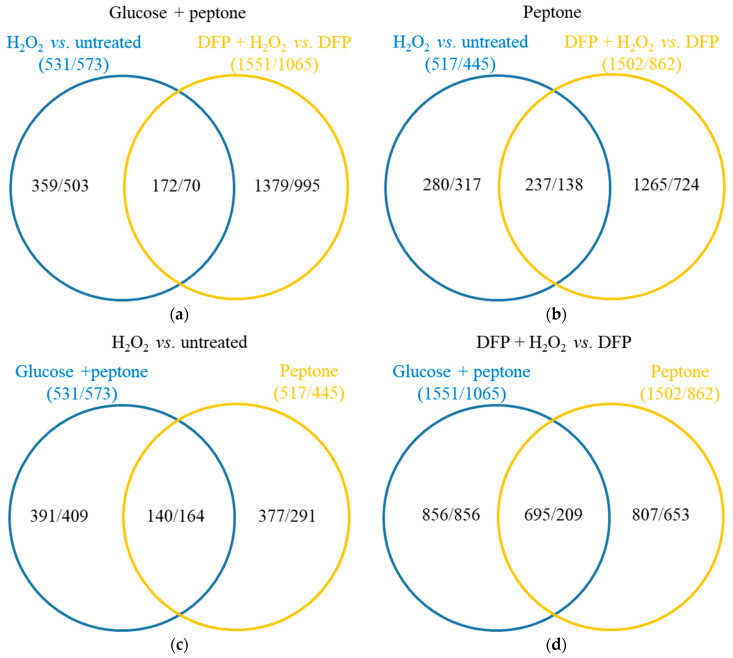
Differences between stress responses elicited by H_2_O_2_. Panels A and B show the Venn analyses of stress-responsive gene sets detected in the absence and in the presence of DFP on glucose + peptone (**a**) or on peptone (**b**). Panels C and D show the Venn analyses of stress-responsive gene sets detected on glucose + peptone and on peptone in the absence (**c**) and in the presence of DFP (**d**). The number of upregulated/downregulated genes are presented.

H_2_O_2_ treatment upregulated 531 and 517 genes, while it downregulated 573 and 455 genes in iron-supplemented cultures in the presence or absence of glucose, respectively (Figure 6). The two types of cultures responded differently to the H_2_O_2_ treatment, as it was suggested by the relatively small overlap between the treatment-responsive gene sets (<50%; Figure S1) and the Pearson’s correlation coefficient between the two transcriptional responses (0.408; Table S1). Regarding iron-limited (DFP treated) cultures, H_2_O_2_ treatment upregulated/downregulated much more genes (1551/1065 on glucose + peptone and 1502/862 on peptone) than in iron-supplemented cultures (Figure 8).

The stress responses of cultures growing on glucose + peptone or peptone differed from each other as in the case of iron supplemented cultures and also differed from those observed with iron-supplemented cultures (Figure 8 and Figure S1, Table S1). Importantly, in the case of cultures growing on peptone, the difference between the two H_2_O_2_ stress responses (observed with iron supplemented or DFP treated cultures) was less pronounced than on glucose + peptone (Figure 8, Table S1). Concurring with the negative results of the DCF-tests (Figure 1b), H_2_O_2_ treatment did not upregulate antioxidative enzyme genes in iron-supplemented cultures (Table S3, Figure 5d). Catalase and peroxidase genes were even enriched in the downregulated gene set by H_2_O_2_ treatment on glucose + peptone (Figure 7, Table S3). Upregulation of only the *cat2* catalase (on glucose + peptone) (Figure 7, Table S3) and a putative thioredoxin gene (Afu5g13640; on peptone) was observed (Table S3). Interestingly, the *aoxA* alternative oxidase gene upregulated both on glucose + peptone and peptone supporting the view that this protein helps maintain the redox status of the mitochondria [42,43,44]. H_2_O_2_ treatment, however, altered iron metabolism (Table S3). In the presence of glucose, the Siderophore cluster genes and Fe-S cluster protein genes were enriched in the upregulated gene set together with TCA cycle genes, while iron-acquisition genes and heme binding protein genes were enriched in the downregulated gene set (Table S3). In the absence of glucose, both Siderophore cluster genes and iron-acquisition genes were enriched in the upregulated gene set. H_2_O_2_ treatment altered the transcription of secondary metabolite cluster genes (Table S3). Besides the upregulation of Siderophore cluster genes, downregulation of 11 clusters (including DHN-melanin cluster) were observed on glucose (Table S3). In the absence of glucose, six clusters showed upregulation (including the mentioned Siderophore cluster) and seven showed downregulation (including DHN-melanin and gliotoxin clusters) (Table S3). Enrichment of amino acid biosynthesis genes in the upregulated gene set on glucose + peptone and glutamine family amino acid catabolic genes in the downregulated gene set on peptone are also notable (Table S2).

The H_2_O_2_ treatment of DFP-pretreated cultures enriched antioxidative enzyme genes in the upregulated gene set (Table S3), concurring well with the increased redox imbalance detected with the DCF test (Figure 1b). In the case of glucose + peptone cultures, only “thioredoxin, glutaredoxin, glutathione system” genes, while for peptone, both “thioredoxin, glutaredoxin, glutathione system” and “catalase and peroxidase” genes, showed enrichment (Table S3, Figure 7). Basically, besides the presence of glucose, the combined DFP + H_2_O_2_ treatment caused substantial transcriptional changes in the antioxidative enzyme gene set (Figure 5d). The upregulations were more prominent on peptone than on glucose + peptone and as a consequence antioxidative enzyme genes were enriched in the upregulated gene set when the transcriptome of the DFP + H_2_O_2_-treated cultures were compared (glucose + peptone, DFP + H_2_O_2_ vs. peptone, DFP + H_2_O_2_) (Table S3). Nevertheless, *yap1* and *atfA* encoding transcription factors involved in the regulation of oxidative stress response [45,46] were also upregulated by H_2_O_2_ treatment of DFP-pretreated cultures on peptone (Table S3). Fe-S cluster assembly genes were enriched in the upregulated gene set in the presence of glucose, while Fe-S cluster assembly and Fe-S cluster protein genes were enriched in the upregulated gene set, and ergosterol biosynthesis genes and heme binding protein genes (other than catalase and peroxidase genes) were enriched in the downregulated gene set (Table S3). In accordance with the reduced growth of DFP + H_2_O_2_-treated cultures (Figure 1a), several processes related to vegetative growth showed enrichment in the downregulated gene set (Table S2). On glucose + peptone, “Mitotic cell cycle” and “Cell wall biogenesis” genes, while on peptone, “Cytosolic ribosome”, “Membrane lipid biosynthetic process” and “Amino acid transmembrane transporter activity” genes, are notable (Table S2). Interestingly, H_2_O_2_ treatment resulted in the upregulation of several glycolytic (on glucose +peptone) and copper homeostasis (both on glucose + peptone and peptone) as well as protein refolding and protein ubiquitination genes (on peptone) (Tables S2 and S3).

## 4. Discussion

For efficient infection, pathogens have to adapt to the microenvironments within the host organism. Host-elicited oxidative stress is a common stress that microbes have to cope with when they infect plants, insects and mammals, including humans [6,7,47,48,49]. Studying oxidative stress responses of pathogens can help us to understand how they can survive in the hostile environment within the host. This understanding may lead to improved strategies to treat infections. Unfortunately, a response to one stressor can highly depend on culturing conditions and further stressors acting either concomitantly with the main stressor or as a prior effect [29,30,31]. Therefore, understanding how microbes respond to a stress, like oxidative stress, in vivo using experimental data collected from in vitro experiments is challenging: even a small difference between the in vivo and in vitro conditions may substantially modify the stress response [28,29,30]. Since in vivo studies have technical and (in the case of animal/human hosts) ethical limitations [50], studying combinatorial stress responses can be an efficient alternative approach. This means that the stress response is investigated under different culture conditions, both alone and in combination with other stressors. The data collected can help us to gain a broader insight into the stress response and to distinguish between its “obligatory” (important under multiple conditions and therefore potentially important in vivo too) and “facultative” (characteristic to certain conditions only, therefore not necessarily important in vivo) factors.

Here, we studied oxidative stress responses of four different cultures: glucose–iron-rich, glucose-rich–iron-limited, glucose-limited–iron-rich and glucose–iron-limited cultures. The rationale behind this experimental design was that glucose–iron-rich cultures are typically used in laboratory practice, therefore the majority of our knowledge on *A. fumigatus* physiology is based on such cultures. However, the glucose availability in the human body is low (the normal blood glucose concentration is less than 0.1 g/L), and pathogens have to utilize carbon/energy sources other than glucose to survive within the host. Concurring with this, deletion of the *mcsA* gene-encoding methylcitrate synthase involved in amino acid utilization (degradation of propionyl-CoA) decreased the virulence of *A. fumigatus* [51]. In addition to the oxidative attack [6,7], iron removal is another typical host strategy to control microbial activity [52], and iron acquisition in the host is often considered a key factor in microbial pathogenicity [53].

The transcriptomes of the four types of cultures studied in our experiments were different (Figure 3), indicating that the physiology of these cultures should have also been different. Accordingly, cultures differed from each other in their growth (Figure 1a) and oxidative stress sensitivity (Figure 1b and Figure 2). Not surprisingly, cultures responded to the same oxidative stress treatment with different intensities and with different genes (Figure 8, Table S2). Focusing on ROS eliminating enzyme genes, only *cat2* (catalase–peroxidase) and *aoxA* (alternative oxidase) were upregulated in glucose–iron-rich cultures by H_2_O_2_ treatment (Table S3), and only a putative thioredoxin gene (Afu5g13640) and *aoxA* were upregulated in glucose-limited–iron-rich cultures. Thioredoxin, glutaredoxin and glutathione (TGG) systems genes (seven genes) were enriched in the upregulated gene set, and *cat2*, *sod3* (mitochondrial Mn-SOD) and *ccp1* (cytochrome c peroxidase) were also upregulated in glucose-rich–iron-limited cultures (Table S3). While both the iron dependent catalase, peroxidase genes (four genes including *cat2* and *ccp1*) and the iron independent TGG system genes (eight genes) were enriched in the upregulated gene set in glucose–iron-limited cultures (Table S3). As a consequence, the transcriptional profile of the antioxidant enzyme genes in the four different H_2_O_2_-treated cultures were different (Figure 5d). Out of the 16 genes upregulated by H_2_O_2_ treatment, at least in one type of culture, only *cat2* showed upregulation under three different conditions (and none of them under all the four conditions) (Figure 7, Table S3). Interestingly, deletion of *cat2* only attenuated the virulence moderately and only together with Δ*cat1* gene deletion, suggesting that Cat2 mycelial bifunctional catalase–peroxidase is an important element of oxidative stress protection in *A. fumigatus,* but other catalases and peroxidases can partially substitute it [15]. The cytochrome c peroxidase gene *ccp1*, the alternative oxidase gene *aoxA* and elements of thioredoxin–thioredoxin reductase–peroxiredoxin system (including *trxA* thioredoxin, *trr1* thioredoxin reductase and *aspf3* peroxiredoxin genes) are also notable, since they were upregulated in both iron-limited cultures after H_2_O_2_ treatment (Table S3). Among them, gene deletion mutants of the *aspf3*, *trr1* (*trxR*) and *aoxA* were studied. Both Δ*aspf3* and Δ*trxR* mutants exhibited decreased virulence in a mouse model of pulmonary aspergillosis [11,54]. Moreover, Aspf3 was found to be essential during infection because it can compensate for the decreased activity of iron-dependent antioxidant enzymes under the iron-limited conditions within the mammalian host [32]. Deletion of *aoxA* was not accompanied with reduced virulence; however, the deletion of *cycA* (cytochrome c gene) increased AoxA activity and resulted in elevated oxidative stress tolerance and long-term persistence of the mutant in murine lungs [44].

Redox and iron homeostasis are closely linked. Several iron-dependent proteins affect the redox milieu, e.g., catalases and heme peroxidases are important in ROS elimination, while the mitochondrial electron transport chain is one of the most important ROS-generating systems. Nevertheless, iron, as a redox active transient metal, can enhance or even trigger oxidative stress when liberated in excess [55]. Therefore, besides antioxidative enzyme genes, we also focused on iron homeostasis genes. Interestingly, although carbon limitation had a small effect on iron metabolism, and iron limitation (DFP treatment) altered iron metabolism in a very similar manner in glucose-supplemented and glucose-free cultures, the oxidative stress induced transcriptional changes in iron metabolism genes were highly dependent on the presence of glucose and on the availability of iron (Table 2 and Table S3, Figure 5a–c).

In glucose–iron-rich cultures, iron acquisition and heme binding protein genes (including catalase and peroxidase genes) were enriched in the downregulated gene set, while (extracellular) siderophore cluster genes and Fe-S cluster protein genes were enriched in the upregulated gene set. In glucose-limited–iron-rich cultures, iron-acquisition genes (including siderophore-mediated iron transport, but not RIA genes) were upregulated (Table 2 and Table S3). Although the transcription profile of heme and Fe-S cluster binding protein genes was altered (Figure 5a,b), these genes did not enrich in the stress responsive gene sets (Table 2 and Table S3). Besides siderophore cluster genes, Fe-S cluster assembly genes enriched in the upregulated gene set in glucose-rich–iron-limited cultures (Table 2 and Table S3). On the other hand, siderophore cluster genes, Fe-S cluster assembly and Fe-S cluster protein genes were enriched in the upregulated gene set, and heme binding protein genes (excluding catalase and peroxidase genes) enriched in the downregulated gene set, in glucose–iron-limited cultures (Table 2 and Table S3). These changes suggest that oxidative stress rearranged iron uptake and utilization in a condition dependent manner, rather than simply down-regulating them. With respect to iron uptake, we observed a trend that oxidative stress increased the importance of siderophore-mediated iron transport (based on formation of inert Fe^3+^-complexes) compared to RIA (based on the reduction of Fe^3+^) (Table S3). It is remarkable that the Siderophore cluster genes enriched in the upregulated gene set in all the four culturing conditions. It suggests that siderophore-mediated iron uptake is important not only to cope with iron limitation but also helps to survive oxidative stress. It may provide a safe iron supply in an imbalanced redox milieu. Concurring with these data, deletion of *mirB* (TAF-C transporter gene), *sidA* (ornithine monooxygenase gene involved in extra- and intracellular siderophore biosynthesis) were found to be crucial for virulence [56,57], while the absence of active *sidI*, *sidH*, *sidF*, *sidD* (elements of the extracellular siderophore cluster) or *sidC* (element of the intracellular siderophore gene cluster) genes but not *ftrA* (iron permease, element of RIA) also attenuated virulence [40,57,58]. In addition to siderophore metabolism, appropriate activity of Fe-S cluster assembly appears to also be important since Fe-S cluster assembly genes and Fe-S cluster binding protein genes were enriched in the upregulated gene set in three out of the four studied culturing conditions (Table 2 and Table S3). Fe-S cluster proteins are important in, among others, nitrate and sulfate assimilation, TCA cycle, mitochondrial respiration and ROS generation, biotin and lipoic acid synthesis, or even translation and maintaining DNA integrity [59,60,61,62]. Fe-S cluster proteins are important O_2_ and NO sensors in several organisms and also sense iron and regulate iron metabolism [63]. For example, in *Saccharomyces cerevisiae*, Fe-S cluster proteins regulate the activity of low-iron-sensing Aft1 and Aft2 as well as the high-iron-sensing Yap5 transcription factors [64]. Hence, maintaining the appropriate activity of Fe-S cluster proteins can be a key factor for survival in hostile environments and may represent a target of antifungal therapy. Although Fe-S cluster proteins are important for both the host cells and the pathogens, the effect of therapies based on Fe-S cluster assembly inhibition can be selective: providing the necessary activity of Fe-S cluster proteins can be much more challenging for fungi trying to survive under a combined oxidative–iron-limitation stress than for the host cells with sufficient iron supply and in optimal redox milieu.

We concluded that the oxidative stress response of *A. fumigatus* highly depended on culturing conditions, namely glucose and iron availability. Such flexibility of stress responses can be crucial for effective adaptation to adverse environmental conditions that human pathogens must cope with. Due to this flexibility, predicting the behavior of pathogens in vivo using data collected in vitro is challenging. Our results also demonstrated that iron metabolism was important not only to cope with iron-limitation stress but also to handle oxidative stress efficiently. Besides siderophore secretion, Fe-S cluster protein production also appeared crucial for adaptation to oxidative stress especially under iron-limited conditions. Although the pathogens and host cells live together in the same place, their culturing conditions (e.g., iron availability or occurrence of oxidative stress) can be very different. Therefore, inhibition of a universally important biochemical process, like Fe-S cluster assembly, may inhibit the pathogen growth selectively.

## Figures and Tables

**Table 2:** Transcriptional behavior of selected gene groups.

Gene Sets	Peptone vs. Glucose + Peptone	DFP Pretreated vs. Not Pretreated		H_2_O_2_ Treated vs. Untreated			
		Glucose + Peptone	Peptone	Iron Supplemented		DFP Pretreated	
				Glucose + Peptone	Peptone	Glucose + Peptone	Peptone
Iron acquisition	-	upregulated	upregulated	downregulated	upregulated	-	-
RIA *	-	upregulated	upregulated	downregulated	-	-	-
Siderophore cluster	up/downregulated	upregulated	upregulated	upregulated	upregulated	upregulated	upregulated
Fe-S cluster assembly	-	-	-	-	-	upregulated	upregulated
Fe-S cluster protein	-	downregulated	downregulated	upregulated	-	-	upregulated
Heme biosynthesis	-	-	-	-	-	-	-
Heme binding proteins	up/downregulated	downregulated	up/downregulated	downregulated	-	-	downregulated
Catalases and peroxidases	-	downregulated	downregulated	downregulated	-	-	upregulated
TGG * system	-	-	-	-	-	upregulated	upregulated
TCA * cycle	-	downregulated	downregulated	upregulated	-	-	-
Respiration	-	downregulated	-	-	-	-	-
Ergosterol biosynthesis	-	upregulated	upregulated	-	-	-	downregulated

*—RIA: reductive iron assimilation; TGG: thioredoxin, glutaredoxin, glutathione; TCA: tricarboxylic acid.

## Data Availability

The transcriptome data sets are available in the Gene Expression Omnibus database (GEO; http://www.ncbi.nlm.nih.gov/geo/; accessed on 14 March 2024) with the following accession number: GSE256524.

## Supplementary Materials

The following supporting information can be downloaded at: https://www.mdpi.com/article/10.3390/jof10030221/s1, Figure S1: Differences between stress responses elicited by H_2_O_2_, DFP, and DFP + H_2_O_2_ treatments in untreated cultures and by H_2_O_2_ treatment in DFP treated cultures observed in the presence and absence of glucose. The number of upregulated/downregulated genes are presented; Table S1: Correlation between transcriptional changes detected in this and previous studies; Table S2: Results of the gene set enrichment analyses; Table S3: Transcriptional behavior of genes belonging to selected gene groups.

## References

[1] Bongomin F., Gago S., Oladele R.O., Denning D.W. (2017). Global and multi-national prevalence of fungal diseases-estimate precision. J. Fungi.

[2] Dagenais T.R., Keller N.P. (2009). Pathogenesis of *Aspergillus fumigatus* in invasive aspergillosis. Clin. Microbiol. Rev..

[3] Howard S.J., Arendrup M.C. (2011). Acquired antifungal drug resistance in *Aspergillus fumigatus*: Epidemiology and detection. Med. Mycol..

[4] Burks C., Darby A., Gómez Londoño L., Momany M., Brewer M.T. (2021). Azole-resistant *Aspergillus fumigatus* in the environment: Identifying key reservoirs and hotspots of antifungal resistance. PLoS Pathog..

[5] Brown N.A., Goldman G.H. (2016). The contribution of *Aspergillus fumigatus* stress responses to virulence and antifungal resistance. J. Microbiol..

[6] Prüfer S., Weber M., Stein P., Bosmann M., Stassen M., Kreft A., Schild H., Radsak M.P. (2014). Oxidative burst and neutrophil elastase contribute to clearance of *Aspergillus fumigatus* pneumonia in mice. Immunobiology.

[7] Hatinguais R., Pradhan A., Brown G.D., Brown A.J.P., Warris A., Shekhova E. (2021). Mitochondrial reactive oxygen species regulate immune responses of macrophages to *Aspergillus fumigatus*. Front. Immunol..

[8] Warris A., Ballou E.R. (2019). Oxidative responses and fungal infection biology. Semin. Cell Dev. Biol..

[9] Henriet S., Verweij P.E., Holland S.M., Warris A. (2013). Invasive fungal infections in patients with chronic granulomatous disease. Adv. Exp. Med. Biol..

[10] Zhai P., Shi L., Zhong G., Jiang J., Zhou J., Chen X., Dong G., Zhang L., Li R., Song J. (2021). The OxrA protein of *Aspergillus fumigatus* is required for the oxidative stress response and fungal pathogenesis. Appl. Environ. Microbiol..

[11] Hillmann F., Bagramyan K., Straßburger M., Heinekamp T., Hong T.B., Bzymek K.P., Williams J.C., Brakhage A.A., Kalkum M. (2016). The crystal structure of peroxiredoxin asp f3 provides mechanistic insight into oxidative stress resistance and virulence of *Aspergillus fumigatus*. Sci. Rep..

[12] Reeves E.P., Reiber K., Neville C., Scheibner O., Kavanagh K., Doyle S. (2009). A nonribosomal peptide synthetase (Pes1) confers protection against oxidative stress in *Aspergillus fumigatus*. FEBS J..

[13] Tsitsigiannis D.I., Bok J.W., Andes D., Nielsen K.F., Frisvad J.C., Keller N.P. (2005). *Aspergillus* cyclooxygenase-like enzymes are associated with prostaglandin production and virulence. Infect. Immun..

[14] Lambou K., Lamarre C., Beau R., Dufour N., Latge J.P. (2010). Functional analysis of the superoxide dismutase family in *Aspergillus fumigatus*. Mol. Microbiol..

[15] Paris S., Wysong D., Debeaupuis J.P., Shibuya K., Philippe B., Diamond R.D., Latgé J.P. (2003). Catalases of *Aspergillus fumigatus*. Infect. Immun..

[16] Calera J.A., Paris S., Monod M., Hamilton A.J., Debeaupuis J.P., Diaquin M., López-Medrano R., Leal F., Latgé J.P. (1997). Cloning and disruption of the antigenic catalase gene of *Aspergillus fumigatus*. Infect. Immun..

[17] Lamarre C., Ibrahim-Granet O., Du C., Calderone R., Latgé J.P. (2007). Characterization of the SKN7 ortholog of *Aspergillus fumigatus*. Fungal Genet. Biol..

[18] Lessing F., Kniemeyer O., Wozniok I., Loeffler J., Kurzai O., Haertl A., Brakhage A.A. (2007). The *Aspergillus fumigatus* transcriptional regulator AfYap1 represents the major regulator for defense against reactive oxygen intermediates but is dispensable for pathogenicity in an intranasal mouse infection model. Eukaryot. Cell.

[19] Ma Y., Qiao J., Liu W., Wan Z., Wang X., Calderone R., Li R. (2008). The sho1 sensor regulates growth, morphology, and oxidant adaptation in *Aspergillus fumigatus* but is not essential for development of invasive pulmonary aspergillosis. Infect. Immun..

[20] Valiante V., Heinekamp T., Jain R., Härtl A., Brakhage A.A. (2008). The mitogen-activated protein kinase MpkA of *Aspergillus fumigatus* regulates cell wall signaling and oxidative stress response *Fungal Genet*. Biol..

[21] Rocha M.C., Godoy K.F., de Castro P.A., Hori J.I., Bom V.L., Brown N.A., Cunha A.F., Goldman G.H., Malavazi I. (2015). The *Aspergillus fumigatus pkcAG579R* mutant is defective in the activation of the cell wall integrity pathway but is dispensable for virulence in a neutropenic mouse infection model. PLoS ONE.

[22] de Vries R.P., Riley R., Wiebenga A., Aguilar-Osorio G., Amillis S., Uchima C.A., Anderluh G., Asadollahi M., Askin M., Barry K. (2017). Comparative genomics reveals high biological diversity and specific adaptations in the industrially and medically important fungal genus *Aspergillus*. Genome Biol..

[23] Emri T., Sümegi-Győri V.M., Páll K., Gila B.C., Pócsi I. (2022). Effect of the combinatorial iron-chelation and oxidative stress on the growth of *Aspergillus* species. Res. Microbiol..

[24] Desoubeaux G., Cray C. (2018). Animal Models of Aspergillosis. Comp. Med..

[25] Ene I.V., Adya A.K., Wehmeier S., Brand A.C., MacCallum D.M., Gow N.A., Brown A.J. (2012). Host carbon sources modulate cell wall architecture, drug resistance and virulence in a fungal pathogen. Cell Microbiol..

[26] Emri T., Antal K., Gila B., Jónás A.P., Pócsi I. (2022). Stress responses elicited by glucose withdrawal in *Aspergillus fumigatus*. J. Fungi.

[27] He R., Wei P., Odiba A.S., Gao L., Usman S., Gong X., Wang B., Wang L., Jin C., Lu G. (2024). Amino sugars influence *Aspergillus fumigatus* cell wall polysaccharide biosynthesis, and biofilm formation through interfering galactosaminogalactan deacetylation. Carbohydr. Polym..

[28] Kurucz V., Krüger T., Antal K., Dietl A.M., Haas H., Pócsi I., Kniemeyer O., Emri T. (2018). Additional oxidative stress reroutes the global response of *Aspergillus fumigatus* to iron depletion. BMC Genom..

[29] Brown A.J.P., Cowen L.E., di Pietro A., Quinn J. (2017). Stress adaptation. Microbiol. Spectr..

[30] Emri T., Forgács K., Pócsi I. (2022). Biologia futura: Combinatorial stress responses in fungi. Biol. Futur..

[31] Song J., Shi L., Wang S., Wang Y., Zhu Y., Jiang J., Li R. (2022). Acidic/alkaline stress mediates responses to azole drugs and oxidative stress in *Aspergillus fumigatus*. Microbiol. Spectr..

[32] Brantl V., Boysen J.M., Yap A., Golubtsov E., Ruf D., Heinekamp T., Straßburger M., Dichtl K., Haas H., Hillmann F. (2021). Peroxiredoxin Asp f3 is essential for *Aspergillus fumigatus* to overcome iron limitation during infection. mBio.

[33] Barratt R.W., Johnson G.B., Ogata W.N. (1965). Wild-type and mutant stocks of *Aspergillus nidulans*. Genetics.

[34] Emri T., Molnár Z., Pócsi I. (2005). The appearances of autolytic and apoptotic markers are concomitant but differently regulated in carbon-starving *Aspergillus nidulans* cultures. FEMS Microbiol. Lett..

[35] Sámi L., Emri T., Pócsi I. (2001). Autolysis and aging of *Penicillium chrysogenum* cultures under carbon starvation: III: Glutathione metabolism and formation of reactive oxygen species. Mycol. Res..

[36] Chomczynski P.A. (1993). Reagent for the Single-Step Simultaneous Isolation of RNA, DNA and Proteins from Cell and Tissue Samples. Biotechniques.

[37] Kim D., Paggi J.M., Park C., Bennett C., Salzberg S.L. (2019). Graph-based genome alignment and genotyping with HISAT2 and HISAT-genotype. Nat. Biotechnol..

[38] Liao Y., Smyth G.K., Shi W. (2014). featureCounts: An efficient general purpose program for assigning sequence reads to genomic features. Bioinformatics.

[39] Love M.I., Huber W., Anders S. (2014). Moderated estimation of fold change and dispersion for RNA-seq data with DESeq2. Genome Biol..

[40] Schrettl M., Bignell E., Kragl C., Sabiha Y., Loss O., Eisendle M., Wallner A., Arst H.N., Haynes K., Haas H. (2007). Distinct roles for intra- and extracellular siderophores during *Aspergillus fumigatus* infection. PLoS Pathog..

[41] Haas H. (2012). Iron—A key nexus in the virulence of *Aspergillus fumigatus*. Front. Microbiol..

[42] Magnani T., Soriani F.M., Martins V.P., Nascimento A.M., Tudella V.G., Curti C., Uyemura S.A. (2007). Cloning and functional expression of the mitochondrial alternative oxidase of *Aspergillus fumigatus* and its induction by oxidative stress. FEMS Microbiol. Lett..

[43] Magnani T., Soriani F.M., de Paulo Martins V., Policarpo A.C., Sorgi C.A., Faccioli L.H., Curti C., Uyemura S.A. (2008). Silencing of mitochondrial alternative oxidase gene of *Aspergillus fumigatus* enhances reactive oxygen species production and killing of the fungus by macrophages. J. Bioenerg. Biomembr..

[44] Grahl N., Dinamarco T.M., Willger S.D., Goldman G.H., Cramer R.A. (2012). *Aspergillus fumigatus* mitochondrial electron transport chain mediates oxidative stress homeostasis, hypoxia responses and fungal pathogenesis. Mol. Microbiol..

[45] Qiao J., Liu W., Li R. (2010). Truncated *Afyap1* attenuates antifungal susceptibility of *Aspergillus fumigatus* to voriconazole and confers adaptation of the fungus to oxidative stress. Mycopathologia.

[46] Silva L.P., Horta M.A.C., Goldman G.H. (2021). Genetic interactions between *Aspergillus fumigatus* basic leucine zipper (bZIP) transcription factors AtfA, AtfB, AtfC, and AtfD. Front. Fungal Biol..

[47] Fones H., Preston G.M. (2012). Reactive oxygen and oxidative stress tolerance in plant pathogenic *Pseudomonas*. FEMS Microbiol. Lett..

[48] Buchon N., Silverman N., Cherry S. (2014). Immunity in *Drosophila melanogaster*—From microbial recognition to whole-organism physiology. Nat. Rev. Immunol..

[49] Sun J., Xiao S., Xue C. (2023). The tug-of-war on iron between plant and pathogen. Phytopathol. Res..

[50] Allert S., Brunke S., Hube B. (2016). In vivo transcriptional profiling of human pathogenic fungi during infection: Reflecting the real life?. PLoS Pathog..

[51] Ibrahim-Granet O., Dubourdeau M., Latgé J.P., Ave P., Huerre M., Brakhage A.A., Brock M. (2008). Methylcitrate synthase from *Aspergillus fumigatus* is essential for manifestation of invasive aspergillosis. Cell. Microbiol..

[52] Nairz M., Weiss G. (2020). Iron in infection and immunity. Mol. Asp. Med..

[53] Misslinger M., Hortschansky P., Brakhage A.A., Haas H. (2021). Fungal iron homeostasis with a focus on *Aspergillus fumigatus*. Biochim. Biophys. Acta Mol. Cell Res..

[54] Binder J., Shadkchan Y., Osherov N., Krappmann S. (2020). The essential thioredoxin reductase of the human pathogenic mold *Aspergillus fumigatus* is a promising antifungal target. Front. Microbiol..

[55] Dlouhy A.C., Outten C.E. (2013). The iron metallome in eukaryotic organisms. Met. Ions Life Sci..

[56] Aguiar M., Orasch T., Shadkchan Y., Caballero P., Pfister J., Sastré-Velásquez L.E., Gsaller F., Decristoforo C., Osherov N., Haas H. (2022). Uptake of the siderophore triacetylfusarinine C, but not fusarinine C, is crucial for virulence of *Aspergillus fumigatus*. mBio.

[57] Schrettl M., Bignell E., Kragl C., Joechl C., Rogers T., Arst H.N., Haynes K., Haas H. (2004). Siderophore biosynthesis but not reductive iron assimilation is essential for *Aspergillus fumigatus* virulence. J. Exp. Med..

[58] Yasmin S., Alcazar-Fuoli L., Gründlinger M., Puempel T., Cairns T., Blatzer M., Lopez J.F., Grimalt J.O., Bignell E., Haas H. (2012). Mevalonate governs interdependency of ergosterol and siderophore biosyntheses in the fungal pathogen *Aspergillus fumigatus*. Proc. Natl. Acad. Sci. USA.

[59] Beinert H., Holm R.H., Münck E. (1997). Iron–sulfur clusters: Nature’s modular, multipurpose structures. Science.

[60] Stiban J., So M., Kaguni L.S. (2016). Iron-sulfur clusters in mitochondrial metabolism: Multifaceted roles of a simple cofactor. Biochemistry.

[61] Read A.D., Bentley R.E., Archer S.L., Dunham-Snary K.J. (2021). Mitochondrial iron-sulfur clusters: Structure, function, and an emerging role in vascular biology. Redox Biol..

[62] Romero A.M., Martínez-Pastor M.T., Puig S. (2021). Iron in translation: From the beginning to the end. Microorganisms.

[63] Mettert E.L., Kiley P.J. (2015). Fe-S proteins that regulate gene expression. Biochim. Biophys. Acta.

[64] Outten C.E., Albetel A.N. (2013). Iron sensing and regulation in *Saccharomyces cerevisiae*: Ironing out the mechanistic details. Curr. Opin. Microbiol..

