# Supplementary material for: The Oxidative Stress Response Highly Depends on Glucose and Iron Availability in Aspergillus fumigatus
Source: J Fungi (Basel). 2024 Mar 18;10(3):221. doi: 10.3390/jof10030221 (PMC10970741; doi:10.3390/jof10030221)
Supplement: Supplementary file 1 [file jof-10-00221-s001.zip › Figure S1.pptx]

## Slide 1
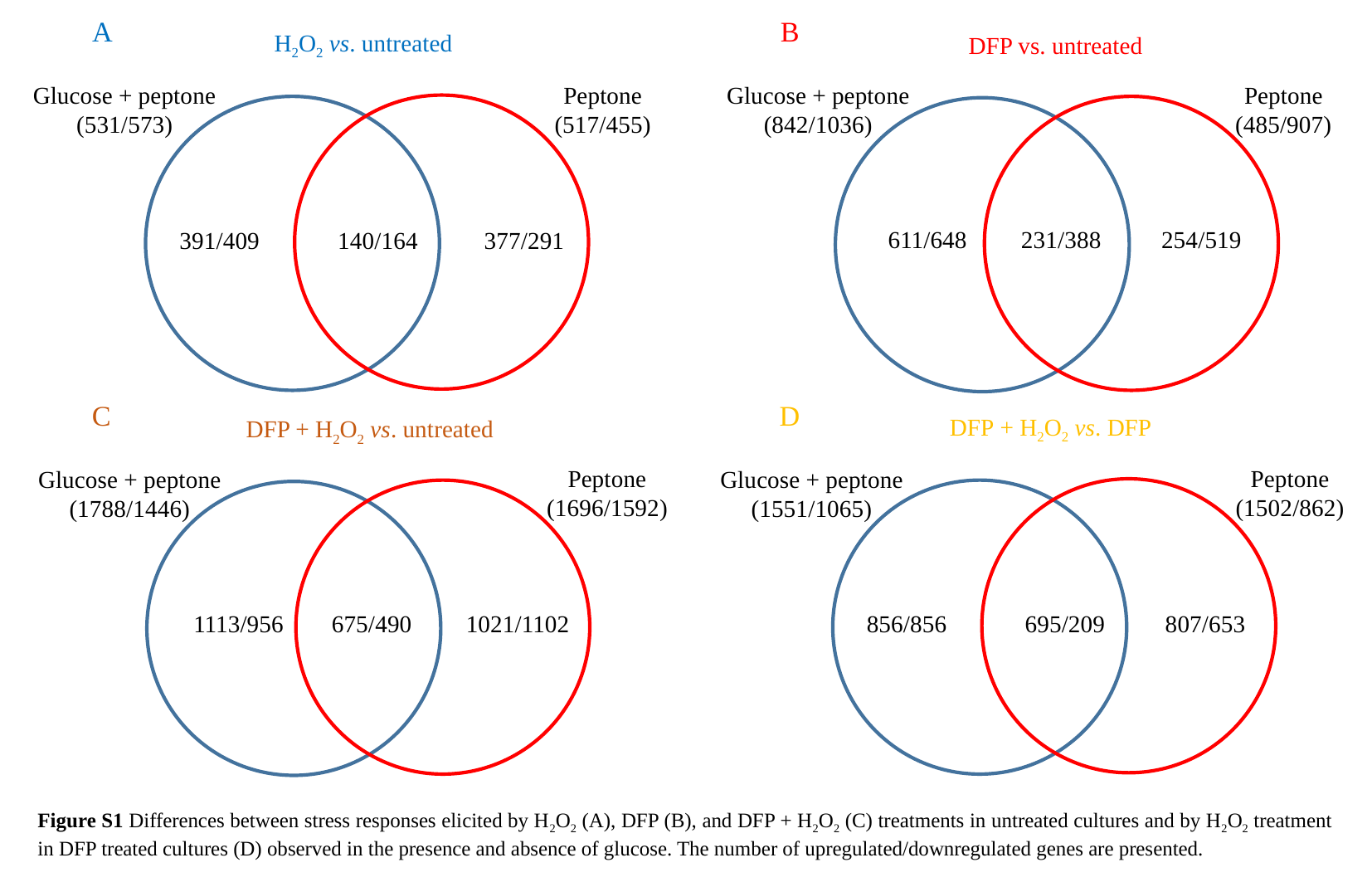

A
B
H2O2 vs. untreated
DFP vs. untreated
Peptone
(517/455)
Peptone
(485/907)
Glucose + peptone
(531/573)
Glucose + peptone
(842/1036)
611/648 231/388 254/519
391/409 140/164 377/291
C
D
DFP + H2O2 vs. DFP
DFP + H2O2 vs. untreated
Peptone
(1696/1592)
Peptone
(1502/862)
Glucose + peptone
(1788/1446)
Glucose + peptone
(1551/1065)
1113/956 675/490 1021/1102
856/856 695/209 807/653
Figure S1 Differences between stress responses elicited by H2O2 (A), DFP (B), and DFP + H2O2 (C) treatments in untreated cultures and by H2O2 treatment in DFP treated cultures (D) observed in the presence and absence of glucose. The number of upregulated/downregulated genes are presented.
